# The Link Between Mothers’ Parental Burnout and Adolescent Aggression: The Roles of Maternal Rejection and Adolescent Empathy

**DOI:** 10.3390/bs15070902

**Published:** 2025-07-03

**Authors:** Qichen Wang, Yuran Qiao, Yanjie Su

**Affiliations:** School of Psychological and Cognitive Sciences, Beijing Key Laboratory of Behavior and Mental Health, Peking University, Beijing 100871, China; wangqichen628@pku.edu.cn (Q.W.); qiaoyuran@stu.pku.edu.cn (Y.Q.)

**Keywords:** parental burnout, aggression, maternal rejection, empathy, gender difference

## Abstract

Although previous studies have shown that parental burnout is a risk factor for adolescents’ development, much remains unknown about the associations between mothers’ parental burnout and adolescents’ aggression as well as the potential mechanisms underlying this relation. To fill these gaps, the current study tested the relationship between mothers’ parental burnout and adolescents’ aggression, as well as the mediating role of maternal rejection and the moderating role of adolescent empathy and gender. A total of 578 Chinese adolescent–mother dyads (for adolescents, 52.42% girls, *M*_age_ = 15.30, *SD* = 1.67; for mothers, *M*_age_ = 42.71, *SD* = 5.49) completed questionnaires regarding mothers’ parental burnout, adolescent aggression, and perceived maternal rejection, as well as empathy. The results showed that mothers’ parental burnout was significantly and positively associated with adolescent aggression and that this relationship was partially mediated by adolescent-perceived maternal rejection. Furthermore, the moderated mediation analysis further revealed that adolescents’ cognitive empathy served as a buffer in the relationship between parental burnout and adolescents’ aggression. In addition, the relation between parental burnout and aggression as well as parental burnout and maternal rejection was stronger for boys. These findings emphasize the need to improve social cognitive abilities in aggression intervention programs.

## 1. Introduction

Parenting is one of the most energy-consuming activities which may lead to stress and exhaustion (Ren et al., 2025). When overwhelming parenting stress and parental coping resources stay imbalanced for a long time, parents are at risk of parental burnout (Mikolajczak & Roskam, 2018; Mikolajczak et al., 2019; Roskam et al., 2017). Parental burnout, typically characterized by parents’ feelings of exhaustion in terms of their parenting role or sudden negative changes in their parental attitude, has begun to receive increasing scholarly attention in recent years (Roskam et al., 2018). In most families, the responsibility for taking care of children primarily falls on mothers (Bianchi & Milkie, 2010; Blanchard et al., 2025), which increases their susceptibility to parental burnout (de Santis et al., 2025). Existing research has demonstrated the negative impacts of parental burnout not only on parents themselves (e.g., sleep disorders, suicidal ideations, and low marital satisfaction; Hormozi et al., 2022; Mikolajczak et al., 2018, 2019; Xu et al., 2024) but also on the developmental outcomes of adolescents (e.g., mental health, adaptation, and social behaviors, Chen et al., 2022; Song et al., 2024; B. Yang et al., 2021).

Adolescence is a period where individuals are prone to showing emotional irritability (Poznanski et al., 2018). Aggression, as a byproduct of emotional irritability, is likely to increase during adolescence (Fagan, 2020). Burned-out mothers may express their exhaustion by rejecting their children’s needs during parent–child interactions (Chen et al., 2022; Song et al., 2024). Consequently, adolescent children are likely to learn and adopt this interaction pattern in their own social relationships, potentially leading to more aggressive behavior toward their peers (Bandura, 1978; W. Li et al., 2022). Thus, more research on the detrimental effects of mothers’ parental burnout on adolescent aggression is theoretically and practically crucial.

Especially in the Chinese sociocultural context, fathers are often viewed as figures of authority and discipline who guide moral development (Short et al., 2001), while mothers are typically regarded as the primary caregivers in the family and usually take on the central role in attending to children’s daily emotional and physical needs (Gaumon & Paquette, 2013). Due to this caregiving structure, Chinese mothers are more susceptible to experiencing parental burnout. Furthermore, Chinese parent–child relationships tend to remain close and interdependent even during adolescence, unlike the more independence-oriented dynamics seen in many Western cultures (Cheung et al., 2016). As such, adolescents in China may be more sensitive to their mothers’ emotional states and parenting behaviors. This highlights the importance of examining how maternal parental burnout influences adolescent outcomes, particularly through the mediating role of maternal rejection, within this cultural setting. The current study aimed to investigate the effects of mothers’ parental burnout on adolescent aggression and expands on previous studies by examining the mediating effects of maternal rejection on this relationship and the moderating effect of adolescent empathy and gender on this mediation process.

### 1.1. Mothers’ Parental Burnout and Adolescent Aggression

Adolescence represents a critical period for the development of aggressive behavior, during which adverse family factors can substantially influence adolescents’ aggression (Nickerson et al., 2025; Farrington, 1993). According to the frustration–aggression model, individuals who experience frustration—whether stemming from personal circumstances or environmental factors that result in unmet needs—are likely to display aggression (Dollard et al., 1939). Parental burnout is typically characterized by a profound sense of exhaustion related to the parenting role and may manifest as sudden negative changes in parental attitudes (Mikolajczak et al., 2018, 2019). Adolescents interacting with burned-out mothers often struggle to meet their own needs, which can lead to feelings of frustration that may ultimately result in aggression.

Beyond theoretical support, empirical research further substantiates the potential positive relationship between parenting burnout and adolescent aggression. A prior study has found that parenting stress, a critical antecedent of parental burnout, longitudinally predicts more problematic behavior of adolescents (Megahead & Deater-Deckard, 2017). In addition, longitudinal research conducted in Chinese mothers–adolescents indicates that, after controlling for adolescents’ age and gender as well as mothers’ age, mothers’ parental burnout positively predicted adolescents’ externalized problem behavior (Chen et al., 2022). One of the typical manifestations of externalizing behaviors is aggression behavior (Maciel et al., 2025). Although prior empirical studies have suggested that maternal parenting burnout may contribute to behavioral problems in adolescents, most of them predominantly focused on broader externalizing behaviors, such as aggression, rule-breaking, and impulsivity. Consequently, there remains a notable gap in the literature regarding the specific relationship between mothers’ parental burnout and adolescent aggression. Furthermore, mediating factors (such as maternal parenting behavior) and individual difference moderators (including adolescents’ levels of empathy or gender) may also influence the connection between mothers’ parental burnout and the aggression of adolescents. Therefore, this study aims to investigate the effect of parenting burnout on adolescent aggression, as well as the potential mediating and moderating factors involved.

### 1.2. The Mediating Role of Maternal Rejection

Rejection is characterized as a pattern of parenting behavior in which a caregiver consistently fails to respond to their child’s emotional needs, occasionally exhibiting behaviors such as punishment or anger (Rohner et al., 2005). Drawing upon Bandura’s social learning theory (Bandura, 1978), maternal rejection cultivates in adolescent aggression through vicarious reinforcement and modeling processes. Adolescents, neurologically primed for social observation (Blakemore & Mills, 2014), are particularly susceptible to internalizing rejecting behaviors as normative relational templates. When caregivers persistently exhibit emotional withdrawal, or even hostility, adolescents may imitate these coercive interaction patterns during peer conflicts and develop an outcome expectation that aggression ensures social control (Bandura, 1978). In line with this theory, some empirical works have supported the association between parental rejection and adolescent aggression. Previous cross-sectional and longitudinal studies have consistently suggested that the level of parental rejection perceived by children at an earlier time point significantly predicted the extent of their subsequent aggressive behavior (Hale et al., 2008; Docherty et al., 2024). Moreover, another multicultural study that included samples from both Eastern and Western countries found that adolescents’ perceptions of maternal rejection significantly predicted their later externalizing behaviors, which encompass typical aggressive behaviors such as tantrums, bullying, and physical violence (Rothenberg et al., 2022). Furthermore, it has been demonstrated that adolescent-perceived maternal rejection negatively predicted social adaptation, including interpersonal harmony (Song et al., 2024). Aggression resulting from maternal rejection may be one of the factors contributing to the disruption of interpersonal harmony. Theoretical and empirical research, therefore, to some extent, indicates that perceived maternal rejection may be associated with adolescent aggression.

According to the conservation of resources theory, parental burnout can result in rejection in parenting. This theory posits that the primary motivation behind one’s behavior is to safeguard resources such as time or energy. When individuals find that their resources are limited, they may resort to avoidant or negative behaviors to prevent any further resource depletion (Hobfoll, 1989). As mentioned earlier, parental burnout results from the imbalance of limited parental coping resources and overwhelming parenting stress (Mikolajczak et al., 2018, 2019; Ren et al., 2025). Therefore, mothers experiencing parental burnout may conserve their resources by decreasing their responsiveness to their children’s needs (e.g., maternal rejection), ultimately preventing further depletion of their resources. Empirical studies have found that mothers’ parental burnout was longitudinally associated with maternal rejection and harsh parenting perceived by young adolescents six weeks later (Song et al., 2024; Xu et al., 2024). Furthermore, another longitudinal study has also found that mothers reporting parental burnout predicted adolescent-perceived maternal hostility six months later (Chen et al., 2022). Hostility is a concept closely related to rejection, as it reflects unkind behavior exhibited by parents toward their children (Rohner et al., 2005).

Most importantly, some empirical studies have demonstrated the mediating role of maternal rejection in the relationship between parental burnout and adolescent problem behavior. For example, studies have found that mothers’ parental burnout predicted adolescent externalizing behaviors and social maladjustment (Chen et al., 2022; Song et al., 2024). Considering that aggressive behavior is a common manifestation of both externalizing behavior and social maladjustment, it is reasonable to infer that mothers’ parental burnout may be positively associated with maternal rejection, which, in turn, is related to adolescent aggression. Existing research has primarily concentrated on early adolescents, and there is a notable gap in studies examining the protective factors within the relationship between parental burnout and aggression. This study was designed to encompass a wider age range of adolescent participants in order to investigate the mediating role of maternal rejection in the relationship between mothers’ parental burnout and adolescent aggression. Additionally, it aimed to examine the protective and moderating factors that play a role in this dynamic.

### 1.3. The Moderating Role of Adolescent Empathy and Gender

One promising protective factor in the relationship between mothers’ parental burnout and adolescent aggression is adolescent empathy, which has been defined as the personal trait of sharing and understanding others’ emotional states (Decety et al., 2016; de Waal, 2008; Q. Wang et al., 2021). As an umbrella term, empathy can be further divided into two components: The first is affective empathy, which refers to emotional contagion and involves a bottom-up process that allows individuals to vicariously share the emotional experiences of others (Decety et al., 2016; de Waal, 2008). The second is cognitive empathy, characterized as a top-down process that enables individuals to understand the emotions of others (Decety et al., 2016; de Waal, 2008). Individuals with high levels of empathy are prone to resonating with others, which then may mitigate harmful behaviors (Van Noorden et al., 2014; Gao et al., 2023). Additionally, people higher in empathy may better understand others’ emotional states and perspectives, which enhances interpersonal interactions and reduces conflicts (Van Lissa et al., 2017; Boele et al., 2019).

Although mothers experiencing parental burnout may be associated with adolescent aggression through adolescent-perceived maternal rejection, adolescent empathy, especially cognitive empathy, can serve as a buffer against this association. Adolescents with higher cognitive empathy are more likely to accurately understand their parents’ emotional state and the underlying reasons (Welker & Rote, 2023). They can figure out that maternal burnout stems from the exhaustion of taking care of oneself. Thus, they are less likely to interpret maternal burnout-induced behaviors (e.g., emotional withdrawal or indifferent attitude) as rejection. In line with this, a previous study has found that people with higher empathy showed lower hostile attribution bias (Qiu et al., 2024). In addition, adolescent cognitive empathy is positively linked with emotion recognition accuracy and parent–child relationship quality (Bek et al., 2022; Boele et al., 2019; Lui et al., 2016). Moreover, one study involving experimental manipulation has also found that enhancing adolescent cognitive empathy can effectively reduce conflicts with their mothers (Van Lissa et al., 2017). In summary, the aforementioned studies consistently show that cognitive empathy in adolescents enhances their understanding of parents, strengthens parent–child relationships, and alleviates conflicts between them. Thus, compared to adolescents with low cognitive empathy, those with high cognitive empathy are more considerate and better able to understand their mothers’ attitudes and behaviors stemming from burnout, making them less likely to interpret them as maternal rejection. In other words, adolescent cognitive empathy would weaken the effect of mothers’ parental burnout on adolescent aggression through maternal rejection, especially the relation between mothers’ parental burnout and maternal rejection.

Besides the buffer role of cognitive empathy, affective empathy may also play a protective role in the relation between mothers’ parental burnout and adolescent aggression. Previous empirical studies have found that empathy, particularly affective empathy, can reduce aggressive behavior in attackers by fostering emotional resonance with potential targets (Nickerson et al., 2024; Stavrinides et al., 2010). Moreover, one study has found that affective empathy moderated the relationship between low agreeableness and cyberbullying (Gao et al., 2023). Thus, affective empathy may act as a protective factor that mitigates aggression, even in adolescents who possess specific individual risk factors or who are exposed to various environmental risks that can trigger aggressive behavior. In summary, it is reasonable to make the hypotheses that the two components of empathy may play protective roles in the path between mothers’ parental burnout and adolescent aggression, mothers’ parental burnout and adolescent-perceived maternal rejection, and maternal rejection and aggression.

The gender of adolescents may be another moderating factor that affects the relationship between maternal factors and adolescent aggression. A previous study has found that mothers of boys were more inclined to experience parental stress than mothers of girls (Rayce et al., 2020). Moreover, studies have found that boys perceived higher maternal rejection than girls during adolescence (W. Zhang & Wang, 2023). These results indicate that boys may experience elevated levels of maternal parenting burnout, along with a heightened sense of maternal rejection. Thus, the association between mothers’ parental burnout and maternal rejection may be stronger for boys. In addition, according to a meta-analysis, boys tend to show more externalized emotions (such as anger) than girls (Chaplin & Aldao, 2013). This implies that after perceiving mothers’ parental burnout, boys are more likely to externalize frustrations encountered within the family, leading to expressions of anger or aggressive behavior. In line with this, a previous meta-analysis has shown that negative maternal attitude has a stronger relationship with the psychological adjustment of sons than with the psychological adjustment of daughters (Rohner & Khaleque, 2010). Furthermore, the predictive effects of a lack of interpersonal support on deviant behaviors were only significant among boys and not among girls (H. Wang et al., 2023). These studies suggest that exposure to environmental risk factors may be more strongly associated with boys’ aggression than with girls’. Overall, it is reasonable to hypothesize that the direct and indirect relationship between mothers’ parental burnout and adolescent aggression may be stronger in boys.

### 1.4. The Current Study

**Figure 1:**
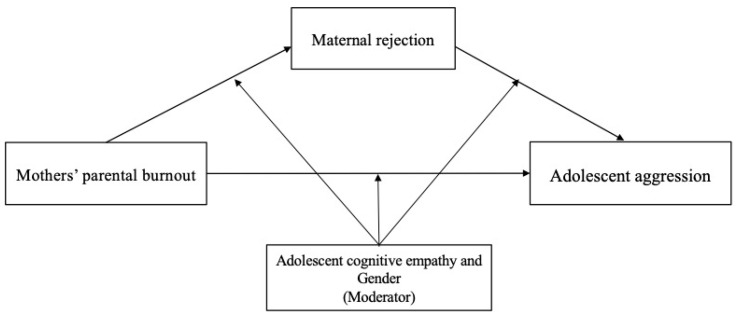
Proposed moderated mediation model between mothers’ parental burnout and regulation effectiveness.

Based on the theoretical and empirical evidence, mothers’ parental burnout is a risk factor for adolescent aggression. However, the mechanisms underlying this link have not been sufficiently studied. According to the frustration–aggression model, social learning theory, and the conservation of resources theory, we considered maternal rejection as a mediator to explain how mothers’ parental burnout would be associated with adolescent aggression. In addition, adolescent empathy and gender may serve as moderators that explain when mothers’ parental burnout would be associated with adolescent aggression. Specifically, the current study tested the mediating effect of maternal rejection on the relationship between mothers’ parental burnout and adolescent aggression (H1) and the moderating effect of adolescent empathy (H2) and gender (H3) on the direct and indirect relationships. These proposed hypotheses in a moderated mediation model are shown in Figure 1.

## 2. Materials and Methods

### 2.1. Participants

A total of 595 adolescent–mother dyads were recruited via an online questionnaire platform from three middle schools in Jiangxi Province, China, in September 2021. After excluding invalid responses, the final sample included 578 adolescents (*M*_age_ = 15.29, *SD* = 1.67, range = 12–19; 52.4% female) and their mothers (*M*_age_ = 42.73, *SD* = 5.44, range = 32–61). All participants provided informed consent, and none reported diagnosed cognitive or sensory disorders. Mothers’ education levels were as follows: 23.5% junior high school or below, 22.4% high school, and 54.1% bachelor’s degree or above.

### 2.2. Measures

#### 2.2.1. Parental Burnout

Mothers reported on their parental burnout using the Parental Burnout Assessment (PBA), which was developed by Roskam, Brianda, and Mikolajczak (Roskam et al., 2018). The PBA has been used globally and has demonstrated validity and reliability among Chinese individuals as well as people from other countries (Cheng et al., 2020; Roskam et al., 2021). The PBA includes 23 items divided into four dimensions: exhaustion in parental role (9 items, e.g., “I have the sense that I’m really worn out as a parent.”); contrast in parental self (6 items, e.g., “I’m no longer proud of myself as a parent.”); feelings of being fed up (5 items, e.g., “I can’t take being a parent anymore.”); and emotional distancing (3 items, e.g., “I’m no longer able to show my child how much I love them.”). Mothers rated each item on a 7-point Likert scale from 0 (never) to 6 (every day). A higher score reflected a higher parental burnout level of adolescent mothers. For the current study, the Cronbach’s α for this scale was 0.93.

#### 2.2.2. Maternal Rejection

Adolescents reported their perceived maternal rejection using the Chinese version of The Egna Minnen av Bamdoms Uppfostran [One’s Memories of Upbringing] (Jiang et al., 2010), which was compiled by Gilbert and Allan (EMBU, Perris et al., 1980). The rejection subscale includes 6 items (e.g., “It happened that my parents gave me more corporal punishment than I deserved.”). Higher scores indicated participants perceiving more maternal rejection. The items are scored on a 4-point Likert scale from 1 (never) to 4 (most of the time). The Cronbach’s α for the rejection subscale in the current study was 0.86.

#### 2.2.3. Adolescent Empathy

Adolescents self-reported their cognitive and affective empathy using the Chinese version of the Griffith Empathy Measure (GEM; Q. Zhang et al., 2014), originally developed by Dadds et al. (2008) for parent ratings. For this study, items were adapted to first-person wording to enable adolescent self-assessment. The reliability and validity of this scale were good in the Chinese adolescent sample (Q. Zhang et al., 2014). The cognitive empathy subscale includes 5 items (e.g., “I rarely understand why other people cry.” [a reverse scoring item]), and the affective empathy subscale includes 6 items (e.g., “ I cry or get upset when seeing another person cry.”). The items are scored on a 9-point Likert scale, from −4 (strongly disagree) to +4 (strongly agree). Higher scores indicate a higher level of the empathic trait. The Cronbach’s α for the cognitive empathy and affective empathy subscales in the current study was 0.81 and 0.86, respectively.

#### 2.2.4. Adolescent Aggression

Adolescent aggression was assessed using the Chinese version of the Buss & Perry Aggression Questionnaire (AQ-CV, X. Li et al., 2011; Buss & Perry, 1992). The Chinese version included 30 items and comprised 5 subscales: physical aggression (7 items, e.g., “If someone hit me, I would fight back.”); verbal aggression (5 items, e.g., “When people disagree with me, I can’t help arguing with them.”); anger (6 items, e.g., “My irritability shows up when things don’t go well.”); hostility (7 items, e.g., “When a stranger is too friendly to me, I suspect that he has another purpose.”), and self-aggression (5 items, e.g., “I hurt myself when I get upset.”). Each item was scored on a scale of 1 (extremely uncharacteristic) to 5 (extremely characteristic). A higher score on the scale was considered more aggressive. The Cronbach’s α in the current sample was 0.94.

### 2.3. Procedure

This study was approved by the Ethics Committee of the authors’ university. Adolescents and their mothers provided informed consent online before accessing the questionnaires. Adolescents completed the anonymous survey independently in school computer rooms, while mothers filled it out at home. Both entered the student number to match responses. The online system required all items to be completed before submission; incomplete responses were excluded. Participation was voluntary, and both parties could withdraw at any time. Anonymized data have been made publicly available in the Open Science Framework and can be accessed at https://osf.io/me9g7/?view_only=4762f52df5484504a72fcf082639688a (accessed on 14 May 2025).

### 2.4. Data Analysis

Data were analyzed using SPSS 28.0 and Mplus 8.3. After standardizing all variables, descriptive statistics and Pearson correlations were conducted in SPSS. Structural equation modeling (SEM) in Mplus 8.3 tested the mediating role of maternal rejection between parental burnout and adolescent aggression, controlling for adolescent gender and age and maternal age (Muthen & Muthen, 2012). Model fit was assessed using *χ*^2^, *df*, TLI, CFI, RMSEA, and SRMR, with standard cutoff criteria (e.g., RMSEA and SRMR ≤ 0.08; CFI and TLI ≥ 0.90). The PROCESS macro (Model 59; Hayes, 2022) was then used to test whether adolescent empathy (particularly cognitive empathy) and gender moderated the mediation effect. To isolate each empathy component’s effect, the other was controlled in the moderation analysis.

## 3. Results

### 3.1. Descriptive Analyses and Bivariate Analyses

Means, standard deviations, and correlations for all variables are presented in Table 1. Mothers’ parental burnout was positively associated with maternal rejection (*r* = 0.28, *p* < 0.001) and adolescent aggression (*r* = 0.19, *p* < 0.001). However, it was not significantly associated with adolescent cognitive empathy (*r* = −0.06, *p* = 0.143) or affective empathy (*r* = −0.01, *p* = 0.780). Additionally, maternal rejection was found to have a positive association with adolescent aggression (*r* = 0.41, *p* < 0.001) and a negative association with both adolescent cognitive empathy (*r* = −0.08, *p* = 0.046) and affective empathy (*r* = −0.12, *p* = 0.004). Furthermore, adolescent aggression did not show a significant association with either adolescent cognitive empathy (*r* = −0.03, *p* = 0.492) or affective empathy (*r* = −0.01, *p* = 0.803).

### 3.2. Test the Mediating Effect of Maternal Rejection

**Figure 2:**
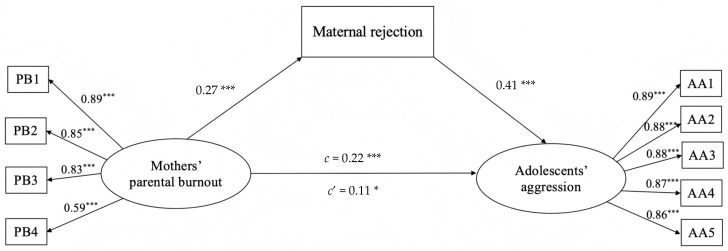
The mediation model of maternal rejection between mothers’ parental burnout and regulation effectiveness. Note. Factor loading is standardized. PB, paternal burnout; AA, adolescents’ aggression. PB1–PB4, four indices of PB; AA1–AA5, five indices of AA. c means the total effect between mothers’ parental burnout and adolescent aggression; c’ means the indirect effect. * *p* < 0.05; *** *p* < 0.001.

Structural equation modeling was used to test whether maternal rejection mediated the association between mothers’ parental burnout and adolescent aggression, while controlling for adolescent gender, adolescent age, and maternal age. Firstly, in the absence of maternal rejection, the direct path coefficient from mothers’ parental burnout to adolescent aggression was significant, with γ = 0.22 and *p* < 0.001. Secondly, a partially mediated model with maternal rejection as a mediator revealed a satisfactory fit to the data: *χ*^2^/*df* = 2.64, RMSEA = 0.05, SRMR = 0.04, CFI = 0.98, and TLI = 0.97. Mothers’ parental burnout was significantly linked to adolescent aggression (β = 0.11, *p* = 0.038) and maternal rejection (β = 0.27, *p* < 0.001). Maternal rejection was significantly linked to adolescent aggression (β = 0.41, *p* < 0.001). That is, mothers’ parental burnout was not only directly associated with adolescent aggression but also indirectly associated with adolescent aggression through maternal rejection. Therefore, in line with the first hypothesis, maternal rejection partially mediated the association between mothers’ parental burnout and adolescent aggression (Figure 2). A bootstrap procedure was employed to evaluate the size of the indirect effect of mothers’ parental burnout on adolescent aggression, along with its confidence intervals (CIs). We generated 5000 bootstrapping samples from the original dataset through random sampling. The indirect effect of maternal rejection was 0.11 (*SE* = 0.03, 95% CI = [0.07, 0.17], *p* < 0.001), accounting for 50.45% of the total effect of the specific path. Moreover, the remaining direct and positive relationship between mothers’ parental burnout and adolescent aggression was still significant (direct effect = 0.11, *SE* = 0.05, 95% CI = [2.07, 0.04]).

### 3.3. Test the Moderating Effects of Adolescent Empathy and Gender

In order to further investigate whether adolescent cognitive empathy and affective empathy would moderate the direct and indirect relationships between mothers’ parental burnout and adolescent aggression, the model 59 in the PROCESS plug-in was run two times. Specifically, we tested whether adolescent cognitive empathy and affective empathy moderated the following: (1) the relationship between mothers’ parental burnout and maternal rejection; (2) the relationship between maternal rejection and adolescent aggression; (3) the relationship between mothers’ parental burnout and adolescent aggression. In each model testing the moderating effect of a specific empathy component, we controlled for another empathy component, as well as the adolescents’ gender and age and maternal age.

Firstly, adolescent cognitive empathy as a moderating variable was brought into the model; as Table 2 illustrates, after controlling for maternal age, adolescents’ gender and age, and adolescent affective empathy, the interaction between mothers’ parental burnout and adolescent cognitive empathy had significant predictive effect on maternal rejection (β = −0.08, *p* = 0.015), but its predictive effect on adolescent aggression was not significant (β = −0.04, *p* = 0.165). The interaction between maternal rejection and adolescent cognitive empathy also had no significant predictive effect on adolescent aggression (β = −0.01, *p* = 0.765).

**Figure 3:**
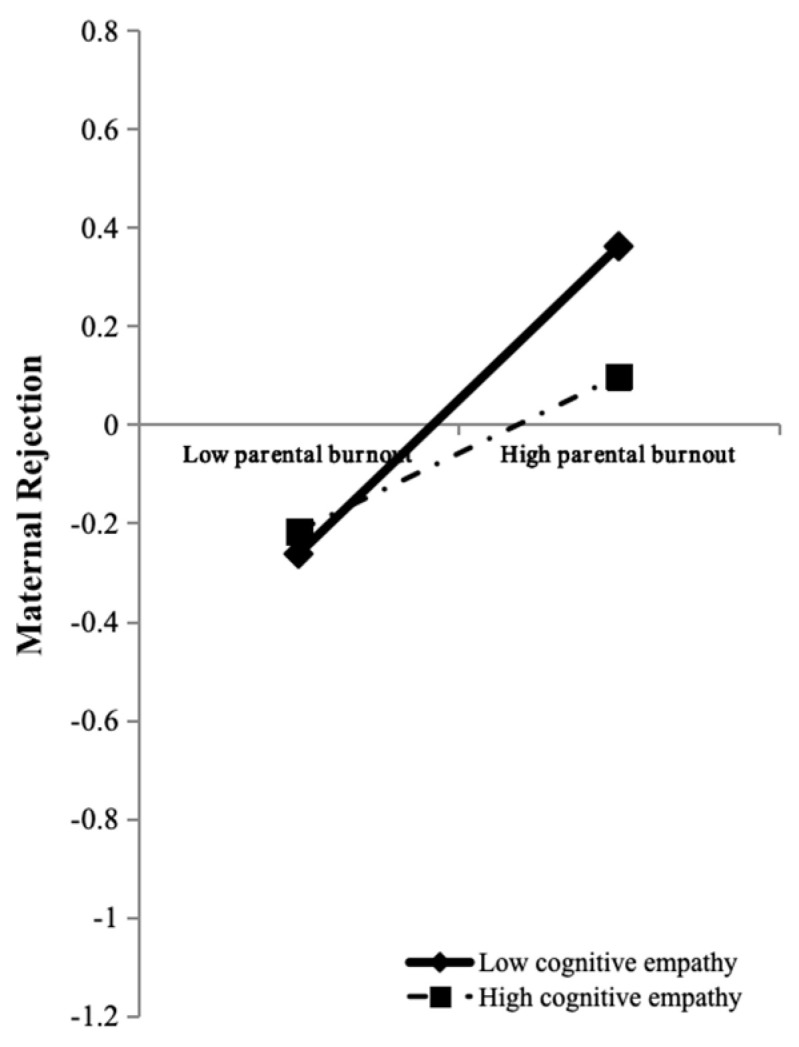
Maternal rejection as a function of mothers’ parental burnout and adolescent cognitive empathy. Note. Functions are graphed for two levels of adolescent cognitive empathy: “high cognitive empathy” means 1 standard deviation above the mean and “low cognitive empathy” means 1 standard deviation below the mean. Note that the variables in the analysis are standardized, and the graph is for descriptive purposes only.

In order to explain the essence of the moderating effect of adolescent cognitive empathy in the path from mothers’ parental burnout to maternal rejection more clearly, simple slope tests were further conducted. The results showed (Figure 3) that for adolescents with lower cognitive empathy, mothers’ parental burnout had significant positive effects on maternal rejection (simple slope = 0.312, *t* = 6.507, *p* < 0.001). For adolescents with higher cognitive empathy, the positive effects of mothers’ parental burnout on maternal rejection were also significant (simple slope = 0.157, *t* = 2.855, *p* = 0.004) but were relatively lower and less significant. This showed that with an improvement in adolescent cognitive empathy, the predictive effect of mothers’ parental burnout on maternal rejection showed downward trend.

When the initial segment of a mediation pathway is influenced by a moderator variable, the overall mediation effect may also be subject to moderation. Accordingly, it is worth examining whether the mediating effect of maternal rejection on the relationship between mothers’ parental burnout and adolescent aggression varies across different levels of adolescent cognitive empathy (the moderator variable). This analysis demonstrated that the mediating effect of maternal rejection on the relationship between mothers’ parental burnout and adolescent aggression was significant among adolescents with lower cognitive empathy (indirect effect = 0.13, 95% CI = [0.06, 0.21]). Meanwhile, the mediation effect was also significant among adolescents with higher cognitive empathy (indirect effect = 0.06, 95% CI = [0.01, 0.12]) but was lower and less significant. Thus, the second hypothesis has been confirmed.

Similarly, we also tested the moderating effect of adolescent affective empathy. As Table 3 illustrates, after controlling for maternal age, adolescents’ gender and age, and adolescent cognitive empathy, the interaction between mothers’ parental burnout and adolescent affective empathy had no significant effect on maternal rejection (β = −0.02, *p* = 0.485) and adolescent aggression (β = 0.04, *p* = 0.271). The interaction between maternal rejection and adolescent affective empathy also had no significant effect on adolescent aggression (β = 0.01, *p* = 0.686). This indicated that the moderating effect of adolescent affective empathy was not significant.

Furthermore, the gender of adolescents as a moderating variable was brought into the model; as Table 4 illustrates, after controlling for adolescent age and maternal age, the interaction between mothers’ parental burnout and the gender of adolescents had a significant effect on maternal rejection (β = −0.11, *p* = 0.004) and adolescent aggression (β = −0.08, *p* = 0.036). Meanwhile, the interaction between maternal rejection and adolescent gender had no significant predictive effect on adolescent aggression (β = 0.05, *p* = 0.200).

**Figure 4:**
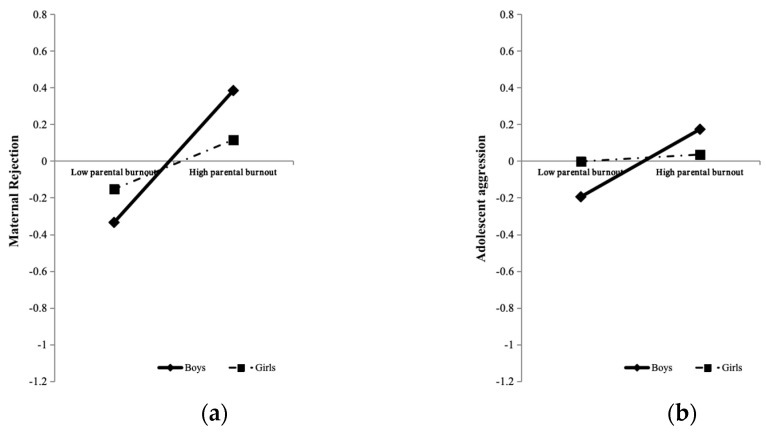
Maternal rejection (**a**) and adolescent aggression (**b**) as a function of mothers’ parental burnout and adolescent gender. Note. The variables in the analysis are standardized, and the graphs are for descriptive purposes only.

In order to explain the essence of the moderating role of adolescent gender more clearly, simple slope tests were further conducted. As for the moderating effect of adolescent gender in the path from mothers’ parental burnout to maternal rejection, the results showed (Figure 4a) that for boys, mothers’ parental burnout had significant positive effects on adolescent-perceived maternal rejection (simple slope = 0.36, *t* = 6.47, *p* < 0.001). For girls, although the positive effects of mothers’ parental burnout on adolescent-perceived maternal rejection were also significant (simple slope = 0.14, *t* = 2.37, *p* = 0.018), they were relatively lower and less significant. Similarly, for the moderating effect of adolescent gender in the path from mothers’ parental burnout to adolescent aggression, the results showed (Figure 4b) that for boys, mothers’ parental burnout had significant positive effects on adolescent aggression (simple slope = 0.18, *t* = 3.32, *p* = 0.001). For girls, the effects of mothers’ parental burnout on aggression were not significant (simple slope = 0.019, *t* = 0.34, *p* = 0.732). This showed that boys are more likely to perceive maternal rejection and engage in cyber-deviant behaviors after experiencing their mothers’ parental burnout. Thus, the third hypothesis has been confirmed.

Additionally, it is important to explore whether the mediating effect of maternal rejection on the relationship between mothers’ parental burnout and adolescent aggression varies between boys and girls. This analysis demonstrated that the mediating effect of maternal rejection on the relationship between mothers’ parental burnout and adolescent aggression was significant among boys (indirect effect = 0.04, 95% CI = [0.06, 0.22]). Meanwhile, the mediation effect was also significant among girls (indirect effect = 0.03, 95% CI = [0.01, 0.13]) but was relatively lower and less significant.

## 4. Discussion

While prior research links maternal parental burnout to adolescent aggression, the underlying mechanisms and protective factors remain unclear. This study examined maternal rejection as a mediator, and adolescent empathy and gender as moderators. The results showed that maternal burnout was positively associated with adolescent aggression, partially through increased maternal rejection. In addition, adolescent cognitive empathy moderated the relation between mothers’ parental burnout and adolescent-perceived maternal rejection. Moreover, the gender of adolescents moderated the association between mothers’ parental burnout and adolescent aggression, as well as mothers’ parental burnout and adolescent-perceived maternal rejection.

### 4.1. The Effect of Mothers’ Parental Burnout on Adolescent Aggression

This study found a positive association between maternal parental burnout and adolescent aggression, suggesting that mothers’ emotional exhaustion may increase adolescents’ risk for aggressive behavior. This aligns with the frustration–aggression model (Dollard et al., 1939), which posits that unmet needs can lead to frustration and subsequent aggression. Burned-out mothers often exhibit emotional exhaustion that affects their caregiving (Mikolajczak et al., 2018, 2019), potentially leading adolescents to experience unmet emotional needs and react with aggression.

Previous longitudinal studies have shown that maternal parental burnout predicts adolescents’ externalizing behaviors and social adjustment issues (Chen et al., 2022; Song et al., 2024). Expanding on this, the present study is the first to examine this link across a broader adolescent age range, focusing specifically on aggression. The findings confirm that maternal burnout remains positively associated with adolescent aggression, even among older adolescents.

### 4.2. The Mediating Effect of Maternal Rejection

This study is the first to identify maternal rejection as a mediator between maternal parental burnout and adolescent aggression. Burned-out mothers may contribute to adolescent aggression through maladaptive parenting. However, since the mediation was only partial, the remaining direct association suggests that parental burnout may also independently heighten adolescent aggression.

Our findings highlight that mothers’ parental burnout is positively linked to maternal rejection, consistent with conservation of resources theory (Hobfoll, 1989), which suggests that individuals facing resource depletion may withdraw to conserve energy. Therefore, mothers experiencing parental burnout may conserve their own energy by reducing their responsiveness to their children’s needs, manifesting as maternal rejection. This link is further supported by longitudinal studies showing associations between mothers’ parental burnout and adolescent-perceived rejection and hostility (Chen et al., 2022; Song et al., 2024; J. Zhang et al., 2025).

In the latter part of the mediation process, maternal rejection was positively associated with adolescent aggression. According to Bandura’s social learning theory, adolescents exposed to maternal rejection may learn to use indifferent or hostile behaviors to achieve goals (Bandura, 1978). This finding is consistent with prior research showing that perceived maternal rejection predicts later externalizing behaviors (Rothenberg et al., 2022). By focusing specifically on adolescent aggression, this study suggests that mothers’ parental burnout is a more fundamental risk factor, indirectly contributing to aggression through rejection perceived by adolescents.

The present findings underscore the unique characteristics of parent–child relationships in Chinese culture, especially the pivotal role of mothers in shaping adolescents’ social behavior development. In China, mothers are typically the primary caregivers responsible for children’s day-to-day physical and emotional needs, while fathers often take on the role of authority figures (Short et al., 2001). As a result, Chinese mothers are more likely to experience high parenting demands and emotional exhaustion, making them particularly vulnerable to parental burnout. Furthermore, Chinese culture emphasizes collectivist values such as familial closeness, obedience, and interdependence (Prioste et al., 2015). Even during adolescence, parent–child relationships remain emotionally close and hierarchical, unlike the increasing independence typically observed in Western cultures. This tight-knit dynamic may increase adolescents’ sensitivity to changes in parenting behaviors. Therefore, when mothers experience parental burnout and exhibit rejecting behaviors, adolescents may be especially affected, leading to heightened emotional and behavioral responses, including aggression. These cultural factors help contextualize the significant mediating role of maternal rejection found in this study and underscore the importance of addressing parental well-being in collectivist societies like China.

### 4.3. The Moderating Effect of Adolescent Cognitive Empathy and Gender

In addition to testing the mediation model, this study examined adolescent empathy as a moderator. The results supported a moderated mediation model, showing that cognitive empathy buffered the indirect link between mothers’ parental burnout and adolescent aggression via perceived maternal rejection. Specifically, parental burnout predicted higher perceived rejection among adolescents low in cognitive empathy, whereas this association was significantly weaker for those with higher cognitive empathy.

The protective role of cognitive empathy lies in its capacity to enhance interpersonal understanding. Defined as empathic perspective-taking, cognitive empathy enables adolescents to grasp others’ emotions and motives by seeing from their perspective (de Waal, 2008). Adolescents with high levels of cognitive empathy are better able to accurately perceive their mother’s state of exhaustion and discern that indifferent parenting behavior just originates from overwhelming childcare fatigue. As a result, they are less likely to interpret parental negative rearing behaviors as rejection. Previous studies have consistently shown that cognitive empathy was positively associated with emotion recognition accuracy and parent–child relationship quality during adolescence (Bek et al., 2022; Boele et al., 2019; Lui et al., 2016). Furthermore, Van der Graaff et al. has also found that parental support predicted less aggression only in adolescents with high empathy (Van der Graaff et al., 2012), suggesting that adolescent empathy may serve as a protective factor against aggression. While Van der Graaff et al.’s study took empathy as a global construct, the current study distinguishes between two sub-components of empathy (cognitive empathy and affective empathy), revealing the specific protective role of cognitive empathy. However, the moderating role of affective empathy was not significant. This may be attributed to the fact that affective empathy tends to develop to a higher level during adolescence (Mestre et al., 2019; Q. Wang et al., 2021), resulting in lower differentiation of affective empathy among adolescents. As a consequence, its protective role in the pathway from parental burnout to aggression is less likely to manifest.

Another noteworthy finding of the current study is that gender moderates the relationship between mothers’ parental burnout and maternal rejection, as well as the relationship between mothers’ parental burnout and adolescent aggression. Specifically, predictive effects were stronger for boys than for girls. This aligns with prior findings that mothers of boys report more parenting stress (Rayce et al., 2020), and adolescent boys perceive more maternal rejection than girls (W. Zhang & Wang, 2023), indicating a gender-biased dynamic in susceptibility to parental burnout and sensitivity to maternal rejection. Furthermore, boys are more likely to express externalizing emotions like anger (Chaplin & Aldao, 2013), making them more prone to responding to maternal burnout with aggression. Supporting this, a meta-analysis found that negative maternal attitudes have a stronger impact on sons’ psychological adjustment than daughters’ (Rohner & Khaleque, 2010), suggesting a gendered pathway from family adversity to behavioral problems.

### 4.4. Limitations and Future Directions

Adopting a dyadic design, this study contributes to the understanding of how mothers’ parental burnout increases the risk of aggression in adolescents and when adolescents’ exposure to parental burnout makes them more likely to exhibit aggression. However, several limitations should be addressed in future research.

First, the data in this study were collected solely through self-report measures from either adolescents or their mothers, which may affect the validity of the findings due to potential biases such as social desirability or inaccurate self-perception (Finnigan & Vazire, 2018). Although the questionnaires used have been validated in previous research, future studies should consider incorporating multi-method approaches, such as behavioral experiments, direct observations, or daily diary methods, to enhance ecological validity and reduce reliance on subjective reporting.

Second, the cross-sectional studies were designed in a manner that inherently limited the ability to interpret causality between the variables. Therefore, it is necessary to investigate how adolescent aggression evolves over time in response to mothers’ parental burnout and associated parenting behaviors through a longitudinal design.

Third, one limitation of the present study is the broad age range of adolescent participants (12 to 19 years). Adolescents at different developmental stages may vary significantly in empathy and social behavior (Q. Wang et al., 2021; Q. Yang et al., 2023). Although we statistically controlled for age in all analyses to reduce its potential confounding effects, future research would benefit from recruiting samples with narrower age ranges to better understand age-specific mechanisms.

Fourth, the current study only investigated the protective factors related to adolescents themselves in the relationship between parental burnout and adolescent aggression. Future research could further explore other protective factors related to mothers, such as mothers’ coping strategies for stress and executive function, which contribute to effective stress regulation (de Santis et al., 2025; Shields et al., 2017).

Fifth, our sample was recruited exclusively from China, which limits the generalizability of the findings to other cultural contexts. For example, in collectivist cultures like China, parent–child relationships tend to be closer and more interdependent (Cheung et al., 2016; Feldman et al., 2010), potentially amplifying the influence of mothers’ parental burnout on adolescent outcomes. Therefore, the conclusions drawn from this study may not fully apply to societies with different parenting norms and family dynamics. Future research should replicate and extend these findings across diverse cultural backgrounds, including both collectivist and individualist societies, to examine whether the observed mechanisms hold universally or are culture-specific.

### 4.5. Implications

Despite the aforementioned limitations, the findings of this study have several theoretical and practical implications. From a theoretical perspective, we expand upon previous research on the risk factors of adolescent aggression by demonstrating that not only a mother’s parenting behavior but also her emotional state is closely related to adolescent aggression. This contribution enriches the existing literature on the mechanisms connecting mothers’ parental burnout with adolescent aggression and highlights related protective factors. To our knowledge, we are the first to show that cognitive empathy and gender moderate both the direct and indirect relationships between mother’s burnout and the aggression of adolescents.

From a practical perspective, the current findings provide valuable insights for designing effective psychological interventions aimed at preventing and reducing adolescent aggression. First, our results highlight mothers’ parental burnout as a significant risk factor for adolescent aggression. This suggests that mental health professionals, school counselors, and social workers should assess not only adolescents’ psychological well-being but also the emotional state and parenting stress levels of their primary caregivers, particularly mothers. Community-based parenting support services and family counseling programs could incorporate burnout screening and offer stress management resources to help mothers cope with parental fatigue.

Second, the finding that the link between parental burnout and adolescent aggression is stronger among boys suggests the need for gender-sensitive interventions. Programs should be tailored to address the specific emotional and behavioral needs of boys, who may be more vulnerable to the effects of maternal rejection and emotional unavailability.

Third, the moderating role of adolescents’ cognitive empathy suggests that enhancing social cognitive skills could serve as a protective factor. School-based social–emotional learning (SEL) programs could incorporate targeted training in cognitive empathy and theory of mind to strengthen adolescents’ interpersonal understanding and reduce aggressive tendencies (Shi & Cheung, 2024). Policymakers and educators might consider integrating such modules into national mental health curricula or extracurricular enrichment programs.

## 5. Conclusions

To summarize, this study makes significant contributions by elucidating the associations between mothers’ parental burnout, parenting behaviors, and adolescents’ social behavior outcomes. Specifically, the present findings provide the first empirical evidence that mothers’ parental burnout is related to adolescents’ perceptions of maternal rejection, which are in turn related to adolescents’ aggression. However, higher cognitive empathy in adolescents weakens the relationship between mothers’ parental burnout and maternal rejection perceived by adolescents. In addition, the predictive effects of mothers’ parental burnout on adolescent-perceived maternal rejection and aggression are much stronger in boys. These findings underscore that exposure to parental burnout may serve as a risk factor for adolescent aggression, particularly among boys, while cognitive empathy may offer a protective buffer against the negative effects of mothers’ parental burnout. This highlights the importance of incorporating caregivers’ feelings of parenting role exhaustion into psychological assessments of adolescents, especially for male adolescents. Furthermore, enhancing social cognitive abilities is essential when developing intervention programs aimed at addressing aggression among adolescents.

## Figures and Tables

**Table 1:** Descriptive statistics and correlations among variables.

	*M*	*SD*	1	2	3	4	5	6	7	8
1. Gender of adolescents	—	—	1							
2. Age of adolescents	15.28	1.65	−0.02	1						
3. Age of mothers	42.73	5.45	−0.02	0.28 ***	1					
4. Mothers’ parental burnout	1.71	0.87	0.03	−0.17 ***	0.02	1				
5. Maternal rejection	1.48	0.52	−0.01	−0.19 ***	−0.19 ***	0.28 ***	1			
6. Adolescent aggression	2.17	0.70	0.01	0.03	0.09 *	0.19 ***	0.41 ***	1		
7. Adolescent cognitive empathy	0.94	1.58	0.10 *	−0.02	0.04	−0.06	−0.08 *	−0.03	1	
8. Adolescent affective empathy	1.01	1.46	0.04	0.17	0.04	0.01	−0.12 **	−0.01	0.15 ***	1

Note. Gender was dummy-coded such that 1 = female and 0 = male. * *p* < 0.05; ** *p* < 0.01; *** *p* < 0.001.

**Table 2:** The moderating effect of adolescent cognitive empathy on the relationship between mothers’ parental burnout and adolescent aggression via maternal rejection.

Predictors	Model 1 (Maternal Rejection)		Model 2 (Adolescent Aggression)	
	β	*t*	β	*t*
Gender of adolescents	−0.01	−0.22	0.01	0.38
Age of adolescents	−0.15	−3.68 **	0.10	2.67 **
Age of mothers	0.03	0.77	0.06	1.72
Adolescent affective empathy	−0.11	0.78	0.02	0.59
Parental burnout	0.23	5.80 ***	0.09	2.14 *
Adolescent cognitive empathy	−0.05	−1.35	0.01	0.21
Parental burnout × Adolescent cognitive empathy	−0.08	−2.42 *	−0.04	−1.38
Maternal rejection			0.40	10.12 ***
Maternal rejection × Adolescent cognitive empathy			−0.01	−0.30

Note. Gender was dummy-coded such that 1 = female and 0 = male. * *p* < 0.05; ** *p* < 0.01; *** *p* < 0.001.

**Table 3:** The moderating effect of adolescent affective empathy on the relationship between mothers’ parental burnout and adolescent aggression via maternal rejection.

Predictors	Model 1 (Maternal Rejection)		Model 2 (Adolescent Aggression)	
	β	*t*	β	*t*
Gender of adolescents	−0.01	−0.27	0.01	0.34
Age of adolescents	−0.15	−3.60 ***	0.11	2.66 **
Age of mothers	0.03	0.71	0.07	1.82
Adolescent cognitive empathy	−0.06	−1.43	0.01	0.23
Parental burnout	0.25	6.19 ***	0.09	2.14 *
Adolescent affective empathy	−0.10	−2.57 ***	0.02	0.57
Parental burnout × Adolescent affective empathy	−0.02	−0.70	0.04	1.10
Maternal rejection			0.42	10.33 ***
Maternal rejection × Adolescent affective empathy			0.01	0.40

Note. Gender was dummy-coded such that 1 = female and 0 = male. * *p* < 0.05; ** *p* < 0.01; *** *p* < 0.001.

**Table 4:** The moderating effect of adolescent gender on the relationship between mothers’ parental burnout and adolescent aggression via maternal rejection.

Predictors	Model 1 (Maternal Rejection)		Model 2 (Adolescent Aggression)	
	β	*t*	β	*t*
Age of adolescents	−0.15	−3.68 **	0.11	2.73 **
Age of mothers	0.02	0.61	0.07	1.78
Parental burnout	0.25	6.15 ***	0.10	2.56 *
Gender of adolescents	−0.02	−0.55	0.01	0.71
Parental burnout × Gender of adolescents	−0.11	−2.85 **	−0.08	−2.10 *
Maternal rejection			0.40	10.01 ***
Maternal rejection × Gender of adolescents			0.05	1.28

Note. Gender was dummy-coded such that 1 = female and 0 = male. * *p* < 0.05; ** *p* < 0.01; *** *p* < 0.001.

## Data Availability

Anonymized data have been made publicly available in the Open Science Framework and can be accessed at https://osf.io/me9g7/?view_only=4762f52df5484504a72fcf082639688a (accessed on 14 May 2025).
